# State Medicaid Coverage for Tobacco Cessation Treatments and Barriers to Coverage — United States, 2008–2014

**Published:** 2014-03-28

**Authors:** Jennifer Singleterry, Zach Jump, Elizabeth Lancet, Stephen Babb, Allison MacNeil, Lei Zhang

**Affiliations:** 1 American Lung Association; 2 Office on Smoking and Health, National Center for Chronic Disease Prevention and Health Promotion, CDC

Medicaid enrollees have a higher smoking prevalence than the general population (30.1% of adult Medicaid enrollees aged <65 years smoke, compared with 18.1% of U.S. adults of all ages), and smoking-related disease is a major contributor to increasing Medicaid costs (1,2). Evidence-based cessation treatments exist, including individual, group, and telephone counseling and seven Food and Drug Administration (FDA)–approved medications (3). A *Healthy People 2020* objective (TU-8) calls for all state Medicaid programs to adopt comprehensive coverage of these treatments.* However, most states do not provide such coverage (4). To monitor trends in state Medicaid cessation coverage, the American Lung Association† collected data on coverage of all evidence-based cessation treatments except telephone counseling§ by state Medicaid programs (for a total of nine treatments), as well as data on barriers to accessing these treatments (such as charging copayments or limiting the number of covered quit attempts) from December 31, 2008, to January 31, 2014. As of 2014, all 50 states and the District of Columbia cover some cessation treatments for at least some Medicaid enrollees, but only seven states cover all nine treatments for all enrollees. Common barriers in 2014 include duration limits (40 states for at least some populations or plans), annual limits (37 states), prior authorization requirements (36 states), and copayments (35 states). Comparing 2008 with 2014, 33 states added treatments to coverage, and 22 states removed treatments from coverage; 26 states removed barriers to accessing treatments, and 29 states added new barriers.¶ The evidence from previous analyses suggests that states could reduce smoking-related morbidity and health-care costs among Medicaid enrollees by providing Medicaid coverage for all evidence-based cessation treatments, removing all barriers to accessing these treatments, promoting the coverage, and monitoring its use (3,5–8).

To assess state Medicaid tobacco cessation coverage, the American Lung Association compiled data through internet searches of websites and documents. Data sources included Medicaid member websites and handbooks, Medicaid provider websites and handbooks, Medicaid policy manuals, and relevant regulations and legislation. Searches were conducted using search functions on Medicaid and other relevant state-sponsored websites and the Google search engine. Researchers searched for mentions of the nine cessation treatments considered in this study. These data were then confirmed through consultations with staff of state Medicaid agencies, staff of state health departments, or other knowledgeable state government personnel. These consultations were also used to supply missing information and reconcile discrepancies. The information on state Medicaid cessation coverage compiled by the American Lung Association has been added to the CDC State Activities Tracking and Evaluation (STATE) System,** a database that contains tobacco-related epidemiologic and economic data and information on state tobacco-related legislation. Although CDC has previously reported data on state Medicaid cessation coverage (4), this is the first time that CDC is reporting information on related barriers.

Comparing 2008 with 2014, 41 states made changes to the treatments they covered for at least some plans or populations, with 19 states adding treatments to coverage without removing any treatments from coverage, eight states removing treatments from coverage without adding any treatments to coverage, and 14 states both adding and removing treatments (Table 1). The treatments most commonly added were individual counseling and the nicotine lozenge; the treatments most commonly dropped were group counseling and the nicotine nasal spray. During this same period, 38 states made changes to barriers to accessing one or more treatments for at least some plans or populations, with nine states removing barriers without adding new barriers, 12 states adding new barriers without removing existing ones, and 17 states both removing and adding barriers (Table 2). The barriers most commonly removed were copayments, duration limits on treatment, and conditioning access to medications on enrolling in counseling; the barriers most commonly added were prior authorization requirements and annual limits. As of 2014, seven states (Connecticut, Indiana, Massachusetts, Minnesota, Nevada, Pennsylvania, and Vermont) cover all nine evidence-based cessation treatments considered in this study for all Medicaid enrollees, with all of these states retaining some barriers to accessing some of these treatments. Also as of 2014, 27 states cover individual counseling and eight states cover group counseling for all populations and plans, whereas 26 states cover all seven FDA-approved cessation medications for all populations and plans. The most common barriers as of 2014 are duration limits (with 40 states reporting this barrier for at least some populations or plans), annual limits (37 states), prior authorization requirements (36 states), and copayments (35 states).

## Discussion

Insurance coverage of evidence-based cessation treatments leads to increases in quit attempts, use of cessation treatments, and successful smoking cessation (3). In particular, more comprehensive state Medicaid coverage for cessation treatments appears to be associated with increased quit rates among smokers enrolled in Medicaid (8). Provisions in coverage that pose barriers to accessing cessation treatments, such as copayments, requirements for prior authorization, and limitations on the number and duration of treatments, might reduce use of these treatments and therefore reduce cessation (3). These provisions are commonly used by private and public health insurers, often to limit use of benefits because of concerns about overuse and resulting costs.†† Removing these barriers would be expected to increase use of cessation treatments and cessation (3,5).

This analysis indicates that although a number of states have added treatments to their state Medicaid cessation coverage and/or removed barriers to accessing treatments during the period 2008–2014, a number of states have removed treatments and/or added new barriers during this period. Although all states now cover some cessation treatments for at least some Medicaid enrollees, only seven states cover all nine treatments considered in this report for all Medicaid enrollees. All seven of these states still have some barriers in place to accessing some of these treatments. Although more states added treatments to coverage than removed treatments from coverage during the study period, more states added barriers to accessing these treatments than removed them.

Several provisions in the 2010 Patient Protection and Affordable Care Act provide opportunities for expanding state Medicaid cessation coverage.§§ Effective October 2010, section 4107 of the Affordable Care Act required state Medicaid programs to cover tobacco cessation counseling and pharmacotherapy for pregnant women with no cost-sharing. This provision resulted in increases in state Medicaid coverage of cessation counseling and medications for pregnant women (9). Additionally, effective January 2014, section 2502 of the Affordable Care Act barred state Medicaid programs from excluding FDA-approved cessation medications from coverage. Although this provision should increase Medicaid enrollees’ access to cessation medications, the extent to which it will do so remains unclear. The impact of the provision will likely depend on how states implement it, and in particular on the extent to which states add cessation medications to preferred drug lists and remove barriers to accessing these medications. The Centers for Medicare and Medicaid Services has issued guidance to states on implementing this provision.¶¶***†††

To obtain a full, accurate assessment of a state’s Medicaid cessation coverage and its impact, it is important to consider, not only the cessation treatments covered and the barriers to accessing those treatments, but the extent to which the state Medicaid program promotes the coverage to smokers enrolled in Medicaid and to health-care providers who serve them and the extent to which the coverage is used. The extent to which Medicaid-covered cessation treatments are actually used plays a key role in determining the impact of cessation coverage, and this is driven by promotion and awareness of the coverage. Studies have suggested that many Medicaid enrollees and many physicians who serve them are not aware of their states’ Medicaid cessation coverage (10) and that, as of 2010, many state Medicaid programs were not promoting their cessation coverage to smokers enrolled in Medicaid (9). Even a cessation benefit that appears comprehensive on paper will have little impact if smokers and health-care providers are unaware of it and do not use it. Conversely, a generous benefit that falls short of being comprehensive might have a substantial positive impact if it is vigorously promoted and widely used. Promoting a cessation benefit to ensure high use might be at least as important an element of comprehensive cessation coverage as covering a specific treatment.

What is already known on this topic?

Medicaid enrollees smoke at a higher rate than the general population, and smoking-related disease is an important contributor to Medicaid costs. Comprehensive state Medicaid cessation coverage has the potential to reduce smoking rates, smoking-related disease, and health-care costs in the Medicaid population. However, previous reports have found that few states provided such coverage.

What is added by this report?

Although progress has been achieved in expanding state Medicaid cessation coverage during 2008–2014, this progress has been mixed. During this period, 33 states added one or more treatments to coverage for at least some plans or populations, whereas 22 states removed treatments from coverage. During this same period, 26 states removed barriers to accessing treatments for at least some plans or populations, compared with 29 states that added at least one new barrier. As of 2014, only seven states cover all nine evidence-based cessation treatments considered in this study for all Medicaid enrollees, and none of these states has removed all barriers to accessing these treatments.

What are the implications for public health practice?

States that cover all evidence-based cessation treatments for all Medicaid enrollees and remove all barriers to accessing these treatments could potentially achieve significant reductions in smoking-related morbidity and health-care costs among Medicaid enrollees. It is also critically important for states to promote their Medicaid cessation coverage to Medicaid smokers and their health-care providers, and to monitor awareness and use of this coverage.

The experience of Massachusetts provides an example of the impact that state Medicaid cessation coverage that is widely promoted can have. An evidence-based cessation benefit was heavily promoted to Medicaid enrollees and their providers, achieving high levels of awareness among Medicaid enrollees (5). Massachusetts used data from the Behavioral Risk Factor Surveillance System to monitor changes in smoking prevalence for Medicaid enrollees and used claims data to monitor use of the cessation benefit (5). The benefit was used by 37% of smokers on Medicaid (approximately 70,000 persons) (5). The benefit was associated with a decrease in the smoking rate among the Medicaid population from 38% to 28% (5), and a nearly 50% reduction in hospital admissions for heart attacks and other acute heart disease diagnoses among smokers who used the benefit (6). The benefit also generated a favorable return on investment: every dollar spent on the benefit was associated with $3.12 in medical savings for cardiovascular conditions alone (7). The Massachusetts example suggests that smokers enrolled in state Medicaid programs are interested in quitting and will take advantage of cessation coverage if this coverage is promoted adequately.

The findings in this report are subject to at least four limitations. First, 2014 data were only partially available for South Dakota. Second, in cases where official documents were not available or conflicted, information on state Medicaid cessation coverage was gathered from knowledgeable state government personnel; this information might have been inaccurate in some cases. Third, cessation coverage can vary widely across Medicaid managed care plans, making it difficult to determine what cessation coverage specific plans provide in practice. Finally, this report does not assess promotion, awareness, or use of state Medicaid cessation coverage. Although examining these factors is essential to accurately evaluate the impact of a state’s Medicaid cessation coverage, the data required to do so are not currently available on an ongoing basis at the national level.

The current status of state Medicaid cessation coverage falls well short of the *Healthy People 2020* target of full coverage in all 50 states and the District of Columbia. States that cover all evidence-based cessation treatments for all Medicaid enrollees and remove barriers to accessing these treatments could substantially reduce smoking rates in a vulnerable population. If states take advantage of its full potential, the provision of the Affordable Care Act that took effect in January 2014 barring state Medicaid programs from excluding cessation medications from coverage might greatly facilitate progress in this regard. States can maximize the impact of their Medicaid cessation coverage by covering counseling as well as medications, promoting their Medicaid cessation benefits, and monitoring awareness and use of these benefits. At present, most states do not appear to be systematically monitoring use of their Medicaid cessation coverage. As indicated by the example from Massachusetts described previously, the fact that most states currently do not provide and promote comprehensive Medicaid cessation coverage is a major missed opportunity to reduce smoking-related morbidity and health-care costs in a population with high smoking rates.

## Figures and Tables

**TABLE 1:** Medicaid coverage for tobacco cessation treatments, by state — United States, 2008 and 2014*†

State	Individual counseling		Group counseling		Nicotine patch		Nicotine gum		Nicotine lozenge		Nicotine nasal spray		Nicotine inhaler		Bupropion (Zyban)		Varenicline (Chantix)	
								
	2008	2014	2008	2014	2008	2014	2008	2014	2008	2014	2008	2014	2008	2014	2008	2014	2008	2014
Alabama	P	P	No	No	No	Yes	No	Yes	No	Yes	No	Yes	No	Yes	No	Yes	No	Yes
Alaska	Yes	Yes	No	No	Yes	Yes	Yes	Yes	Yes	Yes	Yes	No	No	No	Yes	Yes	Yes	Yes
Arizona	No	P	No	No	Yes	Yes	Yes	Yes	Yes	Yes	Yes	Yes	Yes	Yes	Yes	Yes	Yes	Yes
Arkansas	Yes	Yes	Yes	No	Yes	Yes	Yes	Yes	No	No	No	No	No	No	Yes	Yes	Yes	Yes
California	Yes	V	V	V	Yes	Yes	V	Yes	V	Yes	V	Yes	V	Yes	Yes	Yes	V	Yes
Colorado	No	P	No	P	Yes	Yes	Yes	Yes	Yes	Yes	Yes	Yes	Yes	Yes	Yes	Yes	Yes	Yes
Connecticut	No	Yes	No	Yes	No	Yes	No	Yes	No	Yes	No	Yes	No	Yes	No	Yes	No	Yes
Delaware	No	Yes	No	No	Yes	Yes	Yes	Yes	Yes	Yes	Yes	Yes	Yes	Yes	No	Yes	Yes	Yes
District of Columbia	V	Yes	V	No	V	V	V	V	V	V	No	No	No	No	V	No	V	No
Florida	Yes	V	Yes	V	Yes	V	Yes	V	No	V	No	V	No	V	Yes	V	No	V
Georgia	No	Yes	No	No	No	Yes	No	Yes	No	Yes	No	Yes	No	Yes	No	Yes	No	Yes
Hawaii	No	V	V	V	V	Yes	V	Yes	V	V	V	V	V	V	V	V	V	V
Idaho	No	No	Yes	No	Yes	Yes	Yes	Yes	Yes	Yes	Yes	Yes	No	Yes	Yes	Yes	Yes	Yes
Illinois	No	No	No	No	Yes	Yes	Yes	Yes	Yes	Yes	Yes	Yes	Yes	Yes	Yes	Yes	Yes	Yes
Indiana	Yes	Yes	Yes	Yes	Yes	Yes	Yes	Yes	Yes	Yes	Yes	Yes	Yes	Yes	Yes	Yes	Yes	Yes
Iowa	Yes	Yes	No	No	Yes	Yes	Yes	Yes	No	Yes	No	Yes	No	Yes	Yes	Yes	Yes	Yes
Kansas	No	P	No	P	Yes	Yes	No	Yes	No	Yes	No	Yes	No	Yes	Yes	Yes	Yes	Yes
Kentucky	P	V	No	V	No	Yes	No	V	No	V	No	V	No	V	No	V	No	V
Louisiana	No	No	No	V	Yes	Yes	Yes	Yes	No	V	Yes	V	Yes	V	Yes	Yes	Yes	V
Maine	Yes	Yes	No	No	Yes	P	Yes	P	Yes	P	Yes	P	Yes	P	Yes	P	Yes	P
Maryland	Yes	V	Yes	V	V	Yes	V	V	V	V	No	V	No	V	V	Yes	V	V
Massachusetts	Yes	Yes	Yes	Yes	Yes	Yes	Yes	Yes	Yes	Yes	Yes	Yes	Yes	Yes	Yes	Yes	Yes	Yes
Michigan	V	Yes	V	V	Yes	Yes	V	Yes	V	V	V	V	V	V	V	Yes	V	Yes
Minnesota	Yes	Yes	Yes	Yes	Yes	Yes	Yes	Yes	Yes	Yes	Yes	Yes	Yes	Yes	Yes	Yes	Yes	Yes
Mississippi	P	V	P	V	Yes	Yes	Yes	Yes	Yes	Yes	Yes	V	Yes	V	Yes	Yes	Yes	Yes
Missouri	No	Yes	No	No	No	Yes	No	Yes	No	Yes	No	Yes	No	Yes	No	Yes	No	Yes
Montana	Yes	Yes	No	No	Yes	Yes	Yes	Yes	Yes	No	Yes	No	Yes	Yes	Yes	Yes	Yes	Yes
Nebraska	Yes	Yes	Yes	V	Yes	Yes	Yes	Yes	Yes	No	Yes	No	Yes	No	Yes	Yes	Yes	Yes
Nevada	Yes	Yes	Yes	Yes	Yes	Yes	Yes	Yes	Yes	Yes	Yes	Yes	Yes	Yes	Yes	Yes	Yes	Yes
New Hampshire	Yes	Yes	P	P	Yes	Yes	Yes	Yes	Yes	Yes	Yes	Yes	Yes	Yes	Yes	Yes	Yes	Yes
New Jersey	Yes	No	Yes	No	Yes	Yes	V	Yes	No	V	No	V	No	V	V	Yes	V	V
New Mexico	No	V	V	V	V	Yes	V	Yes	V	Yes	V	Yes	V	Yes	V	Yes	V	Yes
New York	P	Yes	P	Yes	Yes	Yes	Yes	Yes	No	V	Yes	V	Yes	V	Yes	Yes	Yes	Yes
North Carolina	No	Yes	No	No	Yes	Yes	Yes	Yes	Yes	Yes	Yes	Yes	Yes	Yes	Yes	Yes	Yes	Yes
North Dakota	Yes	P	Yes	No	Yes	Yes	Yes	Yes	No	Yes	No	Yes	Yes	Yes	Yes	Yes	Yes	Yes
Ohio	No	V	No	V	Yes	Yes	Yes	Yes	Yes	Yes	Yes	Yes	Yes	Yes	Yes	Yes	Yes	Yes
Oklahoma	Yes	Yes	No	No	Yes	Yes	Yes	Yes	Yes	Yes	Yes	Yes	Yes	Yes	Yes	Yes	Yes	Yes
Oregon	Yes	Yes	Yes	V	Yes	Yes	Yes	V	Yes	V	Yes	V	Yes	V	Yes	Yes	Yes	Yes
Pennsylvania	Yes	Yes	Yes	Yes	Yes	Yes	Yes	Yes	Yes	Yes	Yes	Yes	Yes	Yes	Yes	Yes	Yes	Yes
Rhode Island	Yes	Yes	Yes	V	Yes	Yes	Yes	Yes	Yes	Yes	Yes	Yes	Yes	Yes	V	Yes	V	Yes
South Carolina	No	V	No	V	Yes	Yes	Yes	V	Yes	V	Yes	V	Yes	V	Yes	V	Yes	V
South Dakota	No	NA	No	NA	No	P	No	P	No	P	No	No	No	No	Yes	NA	Yes	NA
Tennessee	No	No	No	No	No	Yes	No	Yes	No	Yes	No	Yes	No	Yes	No	Yes	No	Yes
Texas	V	V	V	V	Yes	Yes	Yes	Yes	No	No	Yes	No	Yes	No	Yes	Yes	Yes	Yes
Utah	P	P	P	P	V	V	V	V	V	V	V	V	V	V	Yes	Yes	Yes	Yes
Vermont	No	Yes	No	Yes	Yes	Yes	Yes	Yes	Yes	Yes	Yes	Yes	Yes	Yes	Yes	Yes	Yes	Yes
Virginia	No	Yes	P	V	Yes	Yes	Yes	V	Yes	V	Yes	V	Yes	V	Yes	Yes	Yes	V
Washington	Yes	V	No	No	Yes	V	Yes	V	No	V	No	V	No	V	Yes	V	Yes	V
West Virginia	No	No	V	V	V	Yes	V	Yes	V	Yes	V	Yes	V	Yes	No	Yes	No	No
Wisconsin	Yes	Yes	Yes	V	Yes	Yes	Yes	Yes	No	No	Yes	Yes	Yes	Yes	Yes	Yes	Yes	Yes
Wyoming	Yes	Yes	No	No	Yes	Yes	Yes	Yes	Yes	Yes	No	No	No	No	Yes	Yes	Yes	Yes
Yes	23	27	15	8	38	45	34	40	25	30	28	28	27	29	36	43	35	38
No	20	6	24	20	7	0	8	0	18	5	17	8	18	7	8	1	8	2
Varies by plan (V)	3	11	7	18	6	4	9	9	8	14	6	14	6	14	7	5	8	9
Pregnant women only (P)	5	6	5	4	0	2	0	2	0	2	0	1	0	1	0	1	0	1
Not available (NA)	0	1	0	1	0	0	0	0	0	0	0	0	0	0	0	1	0	1

**Abbreviations:** V = varies by plan; P = pregnant women only; NA = not available.

* Data as of December 31, 2008, and January 31, 2014.

† Because of differences in the methods and timing of data collection, some findings differ from previously reported findings (http://www.cdc.gov/mmwr/preview/mmwrhtml/mm5941a4.htm).

**TABLE 2:** Barriers to Medicaid coverage for tobacco cessation treatments, by state — United States, 2008 and 2014*†§

State	Copayments required		Prior authorization required		Counseling required for medications		Stepped care therapy		Limits on duration		Annual limit on quit attempts		Lifetime limit on quit attempts	
						
	2008	2014	2008	2014	2008	2014	2008	2014	2008	2014	2008	2014	2008	2014
Alabama	No	No	Yes	Yes	NA	Yes	NA	No	Yes	Yes	No	Yes	No	No
Alaska	Yes	Yes	Yes	No	Yes	No	Yes	No	Yes	Yes	Yes	Yes	No	No
Arizona	No	No	No	No	No	No	No	No	Yes	Yes	No	Yes	No	No
Arkansas	No	No	Yes	Yes	Yes	Yes	No	No	Yes	Yes	Yes	Yes	No	No
California	Yes	No	No	V	Yes	V	No	V	Yes	V	Yes	V	No	No
Colorado	Yes	V	Yes	Yes	Yes	V	No	No	Yes	Yes	Yes	Yes	Yes	No
Connecticut	NA	No	NA	Yes	NA	No	NA	No	NA	Yes	NA	Yes	NA	No
Delaware	Yes	Yes	Yes	Yes	Yes	Yes	Yes	Yes	Yes	No	Yes	Yes	No	No
District of Columbia	No	No	No	No	No	No	No	No	Yes	V	No	No	No	No
Florida	Yes	V	No	V	No	V	Yes	V	V	V	V	V	V	V
Georgia	NA	No	NA	Yes	NA	Yes	NA	Yes	NA	Yes	NA	Yes	NA	No
Hawaii	V	V	V	V	V	V	V	V	V	V	V	Yes	V	No
Idaho	No	No	No	Yes	Yes	Yes	No	No	No	No	Yes	Yes	No	No
Illinois	No	Yes	Yes	No	No	No	No	No	No	No	No	No	No	No
Indiana	Yes	Yes	No	No	Yes	Yes	Yes	Yes	Yes	Yes	Yes	Yes	No	No
Iowa	Yes	Yes	Yes	Yes	Yes	Yes	No	Yes	Yes	Yes	Yes	Yes	No	No
Kansas	Yes	No	No	No	No	No	No	No	Yes	Yes	Yes	Yes	No	No
Kentucky	No	No	No	V	NA	V	NA	No	Yes	V	No	V	No	No
Louisiana	Yes	Yes	No	No	Yes	V	No	No	No	V	No	No	No	No
Maine	Yes	No	Yes	Yes	No	No	Yes	Yes	Yes	Yes	Yes	Yes	Yes	Yes
Maryland	V	V	V	V	V	V	V	V	V	V	V	V	V	V
Massachusetts	Yes	Yes	Yes	Yes	No	No	No	No	No	No	No	Yes	No	No
Michigan	V	V	V	V	V	V	V	V	V	V	V	V	V	V
Minnesota	Yes	Yes	No	No	No	No	No	No	No	No	No	No	No	No
Mississippi	Yes	Yes	No	No	No	No	No	No	No	V	No	No	No	No
Missouri	NA	No	NA	Yes	NA	No	NA	No	NA	Yes	NA	No	NA	Yes
Montana	Yes	Yes	Yes	Yes	No	No	Yes	Yes	Yes	Yes	No	Yes	Yes	No
Nebraska	Yes	Yes	Yes	Yes	Yes	Yes	No	No	Yes	Yes	No	Yes	No	No
Nevada	Yes	Yes	Yes	Yes	No	No	No	No	Yes	Yes	Yes	Yes	No	No
New Hampshire	Yes	Yes	Yes	No	No	No	No	No	Yes	No	Yes	Yes	No	No
New Jersey	V	V	V	V	V	No	V	No	V	V	V	V	V	V
New Mexico	No	No	No	V	V	No	No	No	V	V	Yes	V	No	No
New York	V	V	No	V	No	No	No	No	Yes	V	Yes	No	No	No
North Carolina	Yes	Yes	No	No	No	No	No	No	No	No	No	No	No	No
North Dakota	Yes	Yes	Yes	Yes	Yes	Yes	No	No	Yes	Yes	Yes	Yes	No	No
Ohio	Yes	V	No	V	No	No	No	V	No	V	No	No	No	No
Oklahoma	Yes	Yes	Yes	Yes	Yes	Yes	No	No	Yes	Yes	Yes	Yes	No	No
Oregon	Yes	V	No	V	No	V	No	No	No	V	No	V	No	No
Pennsylvania	Yes	Yes	V	V	No	No	No	No	Yes	Yes	Yes	Yes	No	No
Rhode Island	V	No	V	Yes	Yes	Yes	No	Yes	Yes	Yes	No	Yes	No	No
South Carolina	Yes	V	Yes	V	No	V	Yes	V	Yes	Yes	Yes	V	No	No
South Dakota	Yes	Yes	No	No	No	No	No	No	No	No	No	No	No	No
Tennessee	NA	No	NA	Yes	NA	No	NA	No	NA	Yes	NA	No	NA	No
Texas	V	Yes	No	No	V	No	V	No	Yes	No	Yes	No	No	No
Utah	Yes	Yes	Yes	Yes	No	No	No	No	Yes	No	No	No	Yes	No
Vermont	Yes	Yes	No	Yes	Yes	No	No	No	Yes	Yes	Yes	Yes	No	No
Virginia	Yes	V	No	V	No	No	No	V	No	V	No	V	No	No
Washington	No	No	Yes	V	No	V	No	No	No	V	No	V	No	V
West Virginia	No	Yes	Yes	Yes	Yes	Yes	No	Yes	Yes	Yes	No	Yes	No	No
Wisconsin	Yes	Yes	No	No	No	No	No	No	No	No	No	No	No	No
Wyoming	Yes	Yes	No	No	No	No	No	No	Yes	Yes	Yes	Yes	No	No
Yes	30	24	19	21	15	12	7	8	28	24	21	26	4	2
No	10	16	22	15	24	28	33	35	13	11	21	14	38	44
Varies by plan (V)	7	11	6	15	6	11	5	8	6	16	5	11	5	5
Not applicable (NA)	4	0	4	0	6	0	6	0	4	0	4	0	4	0

**Abbreviations:** V = varies by plan; P = pregnant women only; NA = not applicable.

* Data as of December 31, 2008, and January 31, 2014.

† Barriers apply to one or more cessation treatments.

§ Because of differences in the methods and timing of data collection, some findings differ from previously reported findings (http://www.cdc.gov/mmwr/preview/mmwrhtml/mm5941a4.htm).

## Footnotes

* Additional information available at http://www.healthypeople.gov/2020/topicsobjectives2020/objectiveslist.aspx?topicid=41.

† The tobacco use focus area of *Healthy People 2020* recently changed *Healthy People 2020* objective TU-8 to make the American Lung Association (as reported in the STATE System online database) its data source for state Medicaid cessation coverage. CDC had previously used the Center for Health and Public Policy Studies at the University of California, Berkeley as its source for these data. The designated data source for objective TU-8 is being updated to reflect this change.

§ This report assesses state Medicaid coverage of individual counseling, group counseling, and the seven FDA-approved cessation medications. Telephone counseling is available free to callers to state quitlines (including Medicaid enrollees) in all 50 states and the District of Columbia through the national quitline portal 1-800-QUIT-NOW, and therefore is not captured by this report. In June 2011, the Centers for Medicare and Medicaid Services announced that it would offer a 50% federal administrative match to state Medicaid programs for the cost of state quitline counseling provided to Medicaid enrollees.

¶ These changes in coverage and barriers were made for at least some plans or populations. Some states made both positive and negative changes to coverage or barriers during the study period. These states are included in both categories.

** Additional information available at http://www.cdc.gov/tobacco/statesystem. Because of slightly different coding rules and categories, as well as different reporting periods, some data presented in this report differ slightly from Medicaid cessation coverage data reported in the STATE System.

†† Additional information available at http://www.tobaccofreekids.org/press_releases/post/2012_11_26_cessation.

§§ Patient Protection and Affordable Care Act of 2010. Pub. L. No. 114–48 (March 23, 2010), as amended through May 1, 2010. Available at http://docs.house.gov/energycommerce/ppacacon.pdf.

¶¶ Additional information available at http://www.medicaid.gov/medicaid-chip-program-information/by-topics/benefits/prescription-drugs/downloads/rx-releases/state-releases/state-rel-165.pdf.

*** The data on state Medicaid coverage of cessation medications in this report do not reflect this requirement because, as of the writing of this report, state Medicaid programs are still in the process of submitting state plan amendments to bring them into compliance with this provision.

††† In addition to the Affordable Care Act provisions mentioned in this report, this legislation, as written, also provides strong incentives for all states to expand eligibility for Medicaid coverage. Although the Supreme Court ruling in June 2012 held that a state may not lose federal funding for its existing Medicaid program if it chooses not to participate in the expansion, more than half of the states are moving forward with expanding Medicaid at present. This is expected to further increase the number of smokers who have access to cessation treatments in expansion states. However, the information needed to evaluate cessation coverage in the Medicaid expansion population is not currently available.
