# Errata

**Published:** 2014-07-25

## Vol. 63, No. 12

In the report, “State Medicaid Coverage for Tobacco Cessation Treatments and Barriers to Coverage — United States, 2008–2014,” several errors occurred. A listing of the errors and corrections provided by CDC’s Office for Smoking and Health is available online at http://www.cdc.gov/tobacco/data_statistics/mmwrs/byyear/2014/mm6312a3/errata.htm.

